# Diverse mechanisms of bioproduction heterogeneity in fermentation and their control strategies

**DOI:** 10.1093/jimb/kuad033

**Published:** 2023-10-03

**Authors:** Xinyue Mu, Fuzhong Zhang

**Affiliations:** Department of Energy, Environmental and Chemical Engineering, Washington University in St. Louis, St. Louis, MO 63130, USA; Department of Energy, Environmental and Chemical Engineering, Washington University in St. Louis, St. Louis, MO 63130, USA; Division of Biological & Biomedical Sciences, Washington University in St. Louis, St. Louis, MO 63130, USA; Institute of Materials Science & Engineering, Washington University in St. Louis, St. Louis, MO 63130, USA

**Keywords:** Heterogeneity, Fermentation, Bioproduction, Control strategy, Synthetic biology

## Abstract

Microbial bioproduction often faces challenges related to populational heterogeneity, where cells exhibit varying biosynthesis capabilities. Bioproduction heterogeneity can stem from genetic and non-genetic factors, resulting in decreased titer, yield, stability, and reproducibility. Consequently, understanding and controlling bioproduction heterogeneity are crucial for enhancing the economic competitiveness of large-scale biomanufacturing. In this review, we provide a comprehensive overview of current understandings of the various mechanisms underlying bioproduction heterogeneity. Additionally, we examine common strategies for controlling bioproduction heterogeneity based on these mechanisms. By implementing more robust measures to mitigate heterogeneity, we anticipate substantial enhancements in the scalability and stability of bioproduction processes.

**One-sentence summary:**

This review summarizes current understandings of different mechanisms of bioproduction heterogeneity and common control strategies based on these mechanisms.

## Introduction

Microbial cells exhibit inherent variability in morphology (e.g. cell size), growth rate, gene expression, metabolism, and other cellular activities, collectively referred to as microbial heterogeneity. This phenomenon plays a vital role in bacteria persistence, bet-hedging, adaptation to environmental changes, division of labor, and biological diversity, ensuring population-level fitness and survival (Eldar & Elowitz, 2010; Ackermann, 2015; Evans & Zhang, 2020). In the realm of microbial bioproduction, the prevalence of heterogeneity has been extensively documented across diverse bioconversion systems (Mustafi et al., 2012; Pigou & Morchain, 2015; Xiao et al., 2016; Wang et al., 2020; Cao et al., 2021; Bao et al., 2022; Tague et al., 2023).

The impact of heterogeneity on microbial bioproduction is substantial, as it directly affects product quality, yield, process stability, and scalability (Xiao et al., 2016; Rugbjerg et al., 2018). Due to competition among various cellular processes, low- or non-producing cell variants can allocate more resources to growth, which allows them to outcompete high-producing cells and dominate the entire population over time, resulting in diminished productivity and yield (Ceroni et al., 2015). Such a phenomenon becomes particularly problematic in large-scale fermentation, where the extended operation period gives non-producing cells enough time to accumulate (Rugbjerg & Sommer, 2019). Additionally, heterogeneity in microbial bioproduction introduces batch-to-batch variations, posing substantial challenges in obtaining reliable titers and yields. As a result, despite high titer and yield achievements in laboratory-scale microbial production (Peralta-Yahya et al., 2012; Jiang et al., 2018; Bai et al., 2019; Lee et al., 2019), very few have transitioned successfully to industrial manufacturing. Therefore, understanding and controlling heterogeneity in microbial bioproduction are critical for improving the economic competitiveness of the bioproduction industry.

Fortunately, recent studies have been revealing multiple mechanisms that lead to microbial heterogeneity in bioproduction. Breakthroughs in single-cell technologies have contributed to filling knowledge gaps in this complex field. For example, the next-generation sequencing (NGS) enables in-depth analysis of genetic mutations from single cells (Delvigne & Goffin, 2014), while fluorescent-based biosensors help to track single-cell bioproduction as well as to separate high and low producers via cell sorting (Hartline & Zhang, 2022; Zhou & Zhang, 2023). By combining the power of NGS and Fluorescence Activated Cell Sorting (FACS), Sort-seq supports massively parallel reporter assays to quantify the degree of heterogeneity, which greatly expands the throughput of single-cell analysis (Schmitz & Zhang, 2021). Furthermore, machine learning-guided FACS and RNA-seq can reveal community-level heterogeneities in gene expression and metabolic activities (Wang et al., 2023). Many of these tools have been reviewed recently (Delvigne et al., 2015; Evans & Zhang, 2020), and will not be extensively discussed here. In this review, we summarize current insights into different mechanisms of heterogeneity during microbial bioproduction. We then review existing approaches to control heterogeneity based on genetic or non-genetic factors. Finally, we discuss how future technological advances may help further elucidate mechanisms and strategies to improve production robustness.

## Sources of Bioproduction Heterogeneity

**Fig. 1.**
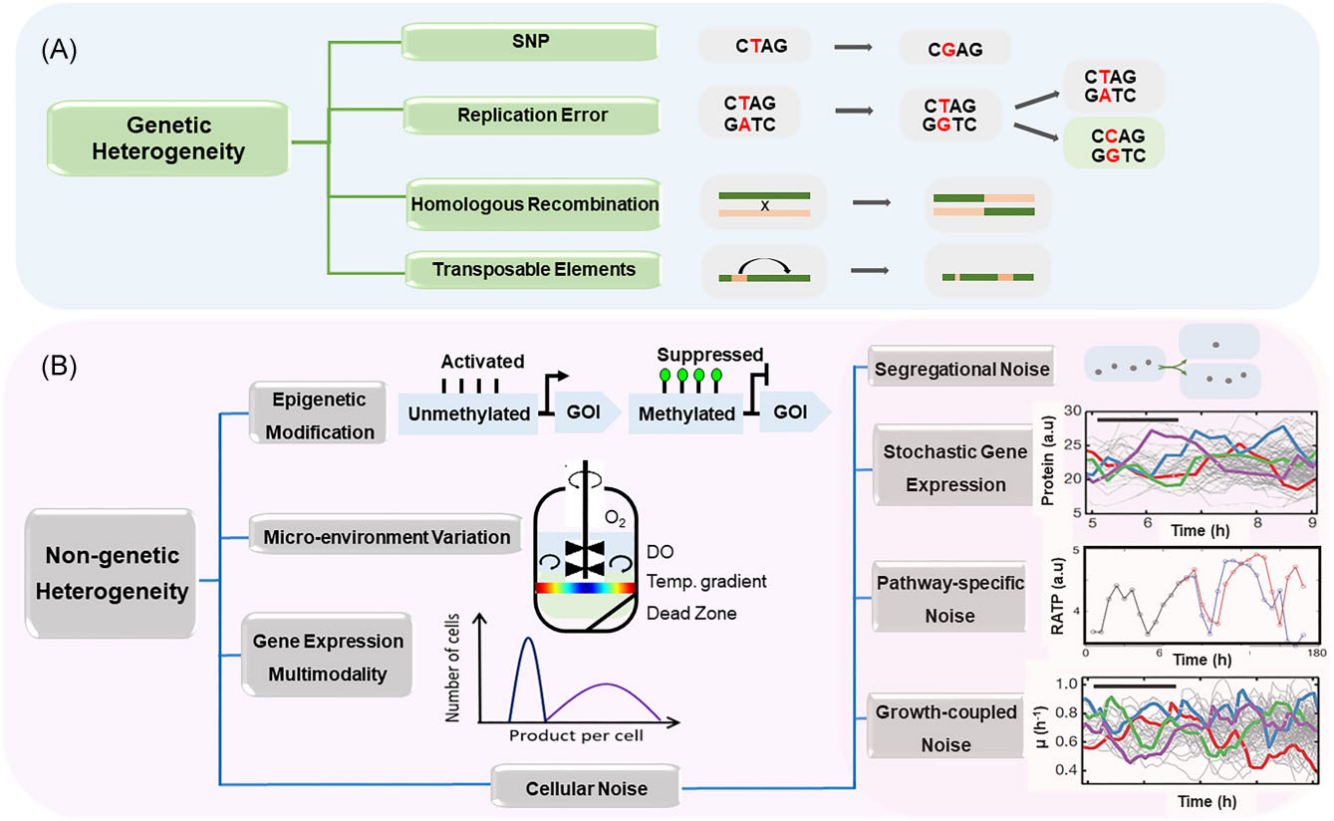
(A) Schematic illustration of the mechanisms leading to genetic heterogeneity. (B) Schematic illustration of the four mechanisms leading to non-genetic heterogeneity. Pathway-specific noise is demonstrated by ATP dynamics in cell division (Lin & Jacobs-Wagner, 2022). Stochastic gene expression is demonstrated by protein abundance over time (Kiviet et al., 2014). Growth-coupled noise is demonstrated by instantaneous growth rate (Kiviet et al., 2014).

Microbial heterogeneity stems from a complex interplay of genetic and non-genetic factors (Fig. 1). Genetic factors refer to diverse genetic mutation mechanisms, including single-nucleotide polymorphism (SNP), replication errors, recombination, and mobile elements such as insertion sequences (ISs) and transposons. Genetic mutations often lead to irreversible consequences in bioproduction. On the other hand, non-genetic factors include epigenetic modifications, variations in micro-environments during fermentation, gene copy number variations, gene expression multimodality, and cellular noise (Binder et al., 2017; Schmitz et al., 2017). These non-genetic factors significantly contribute to variations in metabolism and bioproduction among single cells. Notably, non-genetic variations usually occur at higher frequencies than genetic mutations (see discussions below); thus, they can affect the overall product titer and yield on a shorter timescale than genetic variations. Moreover, non-genetic variations are reversible, meaning that high- and low-producing cells can switch their production states, whereas genetic variations are irreversible (Xiao et al., 2016).

### Genetic Heterogeneity

Rugbjerg and Sommer have provided a comprehensive review that delves into various mechanisms of genetic heterogeneity, encompassing base substitution, homologous recombination, mobile element transposition, and DNA polymerase slippage (Rugbjerg & Sommer, 2019). The impact of genetic heterogeneity on bioproduction was previously discussed (Rugbjerg & Sommer, 2019); hence, it will not be elaborated here. In general, genetic mutation occurs randomly in certain individual cells, causing different bioproduction performances among cells. Under typical growth conditions, genetic mutations occur at seemingly low frequencies. For *Escherichia coli*, the mutation rates range from 10^−5^ per gene per generation for mobile element catalyzed mutation to 10^−10^ per base pair per generation for SNPs. Such frequencies usually start to affect the overall fermentation performance after 50–100 cell doublings, which particularly concerns large-scale bioproduction that requires a series of seed cultures (Rugbjerg et al., 2018). More importantly, the mutation rate can increase substantially under stress conditions during large-scale fermentation (Banerjee & Mukhopadhyay, 2023). Furthermore, the rate of genetic mutation can vary with the host species, biosynthetic pathways, and specific genes depending on their DNA sequences (Binder et al., 2017; Gonzalez-Cabaleiro et al., 2017; Rugbjerg et al., 2021).

The fast-evolving NGS has offered powerful tools for studying rare genetic mutations like SNPs and scars left by IS modifications in large heterogeneous cell populations. Recent application of long-read sequencing has facilitated the investigation of gene tandem amplifications and the discovery of mutation hotspots (Mantere et al., 2019; Xing et al., 2019; De Coster, Weissensteiner, & Sedlazeck 2021). Belikova et al. harnessed long-read sequencing to investigate gene copy number variation in *Staphylococcus aureus.* Their study revealed that the variation of *cas1* gene copy number resulted in heterogeneous immunostimulatory capacities among cells, influencing their host immune response (Belikova et al., 2020). Furthermore, high-throughput technologies enable the simultaneous investigation of multiple mutation factors in a single experiment. Jensen et al. used RNA-seq and transposon sequencing techniques to measure the stress response of three pneumococcal strains under various environmental conditions (Jensen et al., 2017). The results revealed that more than 10% of genes in most functional categories changed their expression as nutrient depleted. Further analysis also indicates a limited correlation between genes governing transcriptional changes and genes impacting overall fitness changes across the entire genome. The combination of sequencing and bioinformatics has empowered precise navigation of mutational activities, providing guidance to eliminate specific targets such as transposable elements.

### Non-genetic Heterogeneity

Non-genetic heterogeneity encompasses a broad range of factors, including epigenetic modification (e.g. DNA methylation), variations in micro-environments, multimodality in gene expression, and cellular noise, which can be further broken down into segregation noise, stochastic gene expression, pathway-specific noise, and growth-coupled noise. In the following sections, we briefly discuss each type of non-genetic heterogeneity.

#### Epigenetic modification

The most common types of epigenetic modification in bacteria are DNA adenine (N6, most prevalent) and cytosine (N4 or N5) methylation (Beaulaurier et al., 2019). In *E. coli*, adenine methylation of GATC sequences in the genome is catalyzed by DNA adenine methyltransferase with limited expression under normal growth conditions. Single cells exhibit distinct methylation patterns, particularly on the hypervariable locus (Cerdeño-Tárraga et al., 2005). These variations can influence gene expression levels if the methylation sites overlap with an upstream regulatory region of a gene (Beaulaurier et al., 2019), causing phenotypic cell-to-cell variations.

#### Variations in micro-environments

Large-scale fermenter inevitably creates heterogeneous micro-environments due to insufficient mixing. Consequently, cells encounter varying local conditions such as temperature, pH, nutrients, and dissolved oxygen (Onyeaka et al., 2003; Lara et al., 2006). A recent study applied a proteomics-based technique to study yeast cells growing in C^13^-labeled glucose and C^12^ lysine. It was found that nutrient gradients resulted in distinct subpopulations of lysine producers and consumers among morphologically undifferentiated yeast colonies. Further, subpopulations underwent fermentative growth, producing and secreting ethanol, which cross-fed other subpopulations of respiring cells (Kamrad et al., 2023).

#### Multi-modality in gene expression

Even when genetically and epigenetically identical cells grow under identical conditions, they still exhibit large phenotypic variations. One mechanism is gene expression multimodality, often driven by positive feedback loops. A well-known example is the arabinose-inducible system, where the arabinose transporter is activated by arabinose, establishing a positive feedback loop (Khlebnikov et al., 2001). At arabinose concentrations ranging from 0.01% to 0.05%, genes controlled by the arabinose-inducible P_BAD_ promoter display bimodality, resulting in phenotypic diversity (Megerle et al., 2008) Therefore, bioproduction of any molecule whose biosynthesis is controlled by a bimodal promoter may have distinct subpopulations with a considerable amount of non-producing cells.

#### Cellular noise

Stochasticity in intracellular bioprocesses gives rise to a universal source of phenotypic heterogeneity known as cellular noise. Cell-to-cell variations in transcription, translation, ATP levels, cofactor abundance, and growth rate have all been widely observed (Raser & O'Shea, 2005; Taniguchi et al., 2010; Paige et al., 2012; Grote et al., 2015; Lin & Jacobs-Wagner, 2022). Further, cellular noise propagates from one molecule or process to another, intricately impacting bioproduction (Kiviet et al., 2014). Here, we briefly discuss different pathways through which cellular noise may affect bioproduction heterogeneity. *Segregation noise*. During cell division, low-copy biomolecules may segregate into daughter cells unevenly, causing different phenotypes between daughter cells. This includes an uneven division of plasmids, low-copy regulators, transporters, and other resources (Bergmiller et al., 2017; Binder et al., 2017). Segregation noise can introduce variability in the expression level of heterologous genes, as well as in substrate uptake rates, leading to the emergence of low-producing subpopulations (Nadell et al., 2010; Perez-Carrasco et al., 2020). *Stochastic gene expression.* Noise in gene expression has been extensively investigated (Engl et al., 2020) with the size of protein noise (squared coefficient of variance, CV^2^) inversely proportional to mean protein abundance until reaching a noise floor (Taniguchi et al., 2010). Although enzymes in bioproduction pathways are usually expressed in high copy numbers, which are associated with relatively low noise values, variation in pathway activities can arise from uncorrelated noise of multiple pathway enzymes. Both experimental and modeling results have demonstrated that due to the competition for intracellular resources, separately expressed proteins are poorly correlated between single cells (Han & Zhang, 2020b). However, coordinating the expression of multiple enzymes from the same mRNA has been proven effective in promoting such correlations (Han & Zhang, 2020b). *Pathway-specific noise*. Some bioproduction pathways are limited by substrate, cofactor, or ATP level. Despite the abundance of small-molecule metabolites typically being orders of magnitude higher than those of proteins, metabolites still exhibit large cell-to-cell variation. For example, ATP noise in *E. coli* reaches 0.62 (CV^2^) (Yaginuma et al., 2014) and varies with growth conditions (Lin & Jacobs-Wagner, 2022). Significant ATP variations may cause heterogeneity in the bioproduction of ATP-limiting products like lipids. *Growth-coupled noise.* Additionally, cell growth rate fluctuates over time and varies among single cells. Noise in growth rate can propagate to cellular processes involved in bioproduction, thus contributing to bioproduction heterogeneity (Kiviet et al., 2014).

During fermentation, microbial heterogeneity often arises from a combination of the aforementioned mechanisms. The degree and the major source of heterogeneity may depend on microbial strains, biosynthetic pathways, choice of promoters and regulation, and fermentation conditions. Notably, the expression of heterologous enzymes or pathways can deplete cellular resources and sometimes accumulates intermediates to toxic levels (Tuite et al., 2005; Jiang et al., 2017; Vincent & Uphoff, 2020). High producers may have higher growth rates than low producers and non-producers, further shaping population dynamics. These distinct mechanisms coupled with evolving population dynamics make it incredibly challenging to pinpoint the primary sources of heterogeneity in bioproduction.

## Strategies to Control Heterogeneity

In pursuit of managing heterogeneity in bioproduction, various strategies have been developed to either reduce the degree of heterogeneity or exploit it for improving bioproduction metrics. In the subsequent sections, we discuss some of these control strategies based on their purposes.

### Reducing Genetic Mutation Rates

**Fig. 2.**
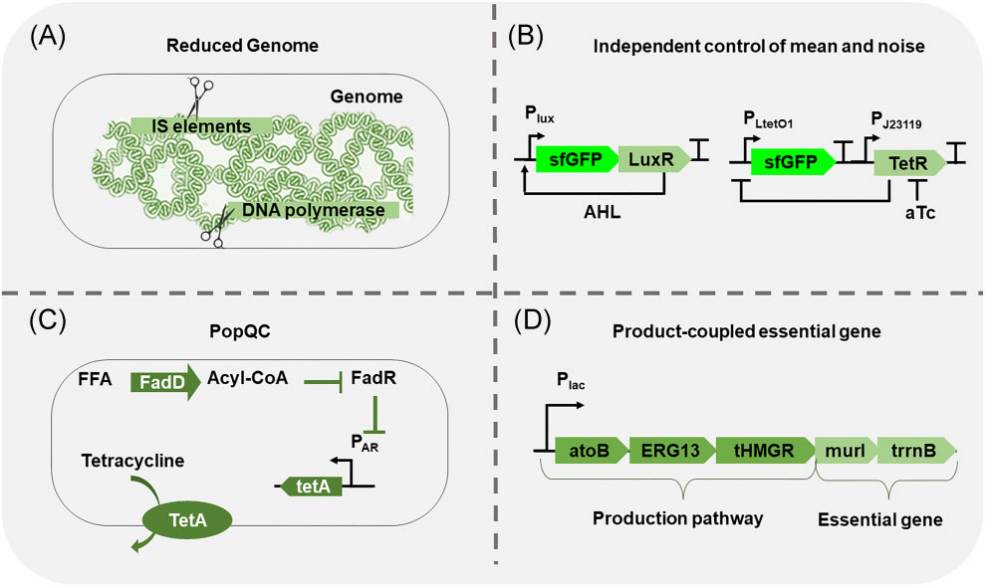
Schematic illustration of control strategies and examples of each strategy. (A) Reducing genetic mutation is demonstrated by deleting IS elements and error-prone DNA polymerases (Csörgő et al., 2012). (B) Reducing gene expression noise is demonstrated by independent control of mean and noise (Gerhardt et al., 2021). (C) Product-based enrichment is demonstrated by PopQC (Xiao et al., 2016). (D) Pathway-based enrichment is demonstrated by coupling essential genes (Rugbjerg et al., 2018).

Genetic heterogeneity can be reduced in a target-specific way once the major mutational mechanism is identified. If transposons and recombinases are the primary sources for mutation, they can be deleted from the host's genome to reduce mutation rates (Fig. 2A) (Csörgő et al., 2012; Nyerges et al., 2019). Likewise, the elimination of IS hotspots from the biosynthetic pathway has been shown to mitigate genetic mutation rates (Choi et al., 2015; Burgard et al., 2016). Furthermore, an *E. coli* strain with reduced genome has been engineered via complete IS deletion (Pósfai et al., 2006). This strain displayed enhanced genetic stability to heterologous gene expression compared to wild-type *E. coli* and has been used for the biosynthesis of multiple products (Pósfai et al., 2006; Lee et al., 2009). Additionally, genome mutation rate can be engineered using directed evolution. Deatherage et al. developed a strategy termed Periodic Reselection for Evolutionarily Reliable Variants (PResERV) (Deatherage et al., 2018), where engineered cells containing a target function are continuously cultivated to accumulate genetic mutations. Periodic sorting is then conducted to isolate cells that retain the original function, thereby identifying mutants that exhibit enhanced stability of the engineered function. PResERV successfully identified several stable *E. coli* strains with improved genetic stability on the engineered function (a gene circuit) alone with a reduced mutation rate (Deatherage et al., 2018).

### Reducing Plasmid Instability

Heterogeneity resulting from plasmid instability often appears during bioproduction. There are several forms of plasmid instability, including plasmid loss, mutation, and copy number variations (Silva et al., 2012). Plasmids that use antibiotic resistance markers to maintain their presence may get lost even in the presence of antibiotic selection (Xu et al., 2006; Chen & Jiang, 2019; Pu et al., 2023). For example, during the production of a phycobiliprotein in engineered *E. coli*, 33% of cells lost their plasmids after 24 h of induction with antibiotic selection present (Chen & Jiang, 2019). Instead of relying on antibiotics, auxotrophic complementation can be used to improve plasmid stability (Singha et al., 2017). However, it is important to note that auxotrophic complementation may cause undesirable growth if the complementation gene is not expressed at the optimal level (Singha et al., 2017). Alternatively, plasmid stability in *E. coli* can be increased by incorporating chromosomal mutations to DNA polymerase I (H734Y) and RNase E (L222S). These chromosomal mutations effectively reduced plasmid mutational rate by 6–30-fold as measured from the Luria–Delbruck fluctuation tests (Rosche & Foster, 2000; Deatherage et al., 2018).

### Reducing Gene Expression Noise

Microbial gene expression is inevitably noisy. For proteins expressed from natural promoters, their noise levels are generally associated with their mean protein abundances. Previous attempts to control gene expression using negative feedback loops have effectively reduced protein noise (Dublanche et al., 2006). Nevertheless, simple feedback loops also substantially reduce mean expression levels, thereby limiting their applications in bioproduction (Dublanche et al., 2006; Han & Zhang, 2020a). Gerhardt et al. developed an elegant approach to independently control protein expression mean and noise levels (Gerhardt et al., 2021). In their strategy, a target protein was expressed from two independent promoters that have high and low noise (Fig. 2B). By tunning the relative strength of each promoter, protein noise and mean abundance can be manipulated independently over a wide expression range in *E. coli*. This approach relies on the use of multiple orthogonal inducible promoters with varying levels of noise generation, which may not be available for certain organisms, or may impose limitations on cost-effective bioproduction in industrial settings. Therefore, there is a pressing need for additional engineering efforts to create inducer-free noise-reduction tools for a wide range of organisms.

### Preventing Gene Expression Multimodality

If bioproduction heterogeneity primarily arises from gene expression multimodality, one approach to mitigate the variations is by removing positive feedback loops involved in the expression system. Early studies on the arabinose-inducible system showed that substituting the arabinose-inducible arabinose transporter with a constitutive promoter effectively eliminated bimodality (Khlebnikov et al., 2001). Alternatively, gene expression can be potentially induced and stabilized in the ON stage even after removing the inducer. Mathematical models have suggested the feasibility of designing an irreversible metabolic switch based on the *E. coli* FadR system (Hartline Christopher et al., 2020; Mannan & Bates, 2021). In this case, the rapid depletion of free FadR upon fatty acid induction initiates the process. Subsequently, the expression of new FadR proteins is repressed by mutual inhibition between FadR and TetR via a positive feedback loop. While the method appears promising in theoretical modeling, it has not yet been experimentally validated. Furthermore, achieving a stable regime is highly dependent on kinetic parameters such as gene expression levels and rates; thus, it would require careful tunning in different microbial hosts. For example, improper parameters may cause cells to deviate from the optimal induced regime.

### Enrichment of High Producers

Bioproduction heterogeneity can also be leveraged to improve overall yield using gene circuits that dynamically select or enrich high producers. Given the dynamic nature of cell growth and bioproduction in fermenters, these strategies must continually adapt. Dynamic metabolic control of cellular metabolism and pathway enzyme expression have been widely applied to optimize pathway activities (Liu & Zhang, 2018; Liu et al., 2018; Hartline et al., 2021). Many of these strategies can also be utilized to select high producers from a heterogeneous population, regardless of the origin of heterogeneity. These strategies can be further categorized as product-based selection or pathway-based selection.

#### Product-based heterogeneity control

An effective approach to enrich high-producing cells from a heterogeneous population (influenced by either genetic or non-genetic factors) is to couple product concentration with the expression of a growth-related gene. The first demonstrated strategy, known as Population Quality Control (PopQC), employs a product biosensor to control the expression of an essential gene (Fig. 2C). Specifically, a free fatty acid (FFA) biosensor was fine-tuned to regulate the expression of a tetracycline efflux pump (Xiao et al., 2016). In this case, only cells producing enough FFA can express a sufficient amount of efflux pump to survive from extracellularly added tetracycline, resulting in a substantial enrichment of high FFA producers, addressing non-genetic variations and resulting in a threefold enhancement in overall FFA titers and yield (Xiao et al., 2016). PopQC proved effective even when the antibiotic survival gene was replaced by essential metabolic genes, such as the leucine biosynthetic pathway, thereby eliminating the dependence of antibiotic selection. Similar designs have been expanded to the production of other compounds in various hosts. For example, a genetic circuit coupling flavonoid biosensor with leucine biosynthesis improved naringenin production in *Yarrowia lipolytica* by 74.8% (Lv et al., 2020). Similarly, growth–production coupling improved *N*-acetylneuraminic acid production in *Bacillus subtilis* by 1.97-fold (Cao et al., 2021) and improved vanillin-β-glucoside production in *Saccharomyces cerevisiae* by twofold (D'Ambrosio et al., 2020). These examples demonstrated that PopQC can effectively enrich high producers from both genetic and non-genetic variants. However, PopQC requires a product biosensor to function, which may not be available for some products.

#### Pathway-based heterogeneity control

A product sensor may be obviated if genetic mutation of the target biosynthetic pathway is the primary heterogeneity mechanism. Rugbjerg et al. demonstrated a simple and effective approach to eliminate non-producing variants resulting from IS disruption of the mevalonate pathway. In this scenario, a growth essential glutamate racemase gene (*murI*) was cloned to the same operon 3′ of the mevalonate pathway (Fig. 2D). The codon shift introduced by IS disruption leads to the silencing of *murI* expression, thereby killing mutated non-producers and maintaining stable bioproduction over 60 cell generations (Rugbjerg et al., 2018).

## Conclusion and Future Perspectives

Addressing bioproduction heterogeneity in fermentation is crucial for optimizing titers, yields, robustness, and scalability. While bioproduction heterogeneity stems from multiple origins, it is critical to identify the major source(s) of variation so that corresponding control strategies can be applied to effectively reduce heterogeneity. Various advanced single-cell analysis technologies, including NGS, cell sorting, biosensors, single-cell imaging, and their combinations, serve as valuable tools for identifying the origins of variation. The continuous development of cutting-edge techniques will make the identification of the heterogeneity source and the underlying mechanisms more efficient.

Once the major source(s) of heterogeneity is identified, proper control tools can be applied. Harnessing the right technology for control is critical in optimizing bioproduction performance. While some tools are tailored to specific mechanisms (e.g. deletion of mobile elements only reduces variation from genetic mutations, and bioproduction will still be heterogeneous due to non-genetic variations), product-based selection tools can address both genetic and non-genetic factors.

Although existing strategies have demonstrated success in various applications, questions remain for further improving fermentation metrics, robustness, and scalability. The efficacy of each control strategy can change depending on several factors such as fermenting time, mutation rates of each control construct, and the response time of the feedback loop. These factors need to be considered case by case for the control strategy to work. Additionally, very few heterogeneity control strategies have been validated and used in industrial practices. Therefore, the development of new strategies is imperative, particularly for large-scale production scenarios characterized by prolonged fermentation periods and a more complex heterogeneous environment. Future heterogeneity control strategies should be tailored to suit the demands of large-scale production. Ultimately, the continued innovation and engineering of these approaches combined with advanced single-cell analysis technology will provide great opportunities for minimizing the effects of cellular heterogeneity and optimizing microbial bioproduction.
